# Age-Related Metabolic Pathways Changes in Dental Follicles: A Pilot Study

**DOI:** 10.3389/froh.2021.677731

**Published:** 2021-06-18

**Authors:** Victor Coutinho Bastos, Jéssica Gardone Vitório, Roberta Rayra Martins-Chaves, Flávia Leite-Lima, Yuri Abner Rocha Lebron, Victor Rezende Moreira, Filipe Fideles Duarte-Andrade, Thaís dos Santos Fontes Pereira, Lucilaine Valéria de Souza Santos, Liséte Celina Lange, Adriana Nori de Macedo, Gisele André Baptista Canuto, Carolina Cavaliéri Gomes, Ricardo Santiago Gomez

**Affiliations:** ^1^Department of Oral Surgery and Pathology, School of Dentistry, Universidade Federal de Minas Gerais, Belo Horizonte, Brazil; ^2^Department of Pathology, Biological Sciences Institute, Universidade Federal de Minas Gerais, Belo Horizonte, Brazil; ^3^Department of Sanitary and Environmental Engineering, School of Engineering, Universidade Federal de Minas Gerais, Belo Horizonte, Brazil; ^4^Department of Chemistry, Exact Sciences Institute, Universidade Federal de Minas Gerais, Belo Horizonte, Brazil; ^5^Department of Analytical Chemistry, Institute of Chemistry, Universidade Federal da Bahia, Salvador, Brazil

**Keywords:** aging, dental follicle, dental sac, developmental biology, oral pathology, untargeted metabolomics, LC-MS

## Abstract

Aging is not a matter of choice; it is our fate. The “time-dependent functional decline that affects most living organisms” is coupled with several alterations in cellular processes, such as cell senescence, epigenetic alterations, genomic instability, stem cell exhaustion, among others. Age-related morphological changes in dental follicles have been investigated for decades, mainly motivated by the fact that cysts and tumors may arise in association with unerupted and/or impacted teeth. The more we understand the physiology of dental follicles, the more we are able to contextualize biological events that can be associated with the occurrence of odontogenic lesions, whose incidence increases with age. Thus, our objective was to assess age-related changes in metabolic pathways of dental follicles associated with unerupted/impacted mandibular third molars from young and adult individuals. For this purpose, a convenience sample of formalin-fixed paraffin-embedded (FFPE) dental follicles from young (<16 y.o., *n* = 13) and adult (>26 y.o., *n* = 7) individuals was selected. Samples were analyzed by high-performance liquid chromatography-mass spectrometry (HPLC-MS)-based untargeted metabolomics. Multivariate and univariate analyses were conducted, and the prediction of altered pathways was performed by *mummichog* and Gene Set Enrichment Analysis (GSEA) approaches. Dental follicles from young and older individuals showed differences in pathways related to C21-steroid hormone biosynthesis, bile acid biosynthesis, galactose metabolism, androgen and estrogen biosynthesis, starch and sucrose metabolism, and lipoate metabolism. We conclude that metabolic pathways differences related to aging were observed between dental follicles from young and adult individuals. Our findings support that similar to other human tissues, dental follicles associated with unerupted tooth show alterations at a metabolic level with aging, which can pave the way for further studies on oral pathology, oral biology, and physiology.

## Introduction

Dental follicles, also referred to as dental sacs, comprise a defined structure with a remarkable role in periodontogenesis and tooth eruption [1–4]. Classically, the definition of dental follicle includes the ectomesenchymal tissue derived from the neural crest that involves the tooth during development (i.e., odontogenesis) [5]. By a surgical definition, however, the term “dental follicle” comprises both the ectomesenchymal tissue around the developing tooth as well as the reduced enamel epithelium [6]. In cases when tooth eruption does not occur, the dental follicle remains attached to the coronal portion of the unerupted or impacted tooth [7]. Some pathological changes are described in this context, and are often age-dependent, as in the case of squamous and mucous cell metaplasia/prosoplasia. They involve molecular changes, such as increased expression of cell proliferation markers and lower expression of pro-apoptotic markers, mainly attributed to the epithelial component of this dental sac [8–12]. Some of these molecular and morphological alterations are considered as early signs of events that give rise to odontogenic lesions, such as developmental cysts and tumors. However, this is known to be a rare event [13].

Aging is a complex process that involves alterations in many cellular processes resulting in/from altered cell-cell interactions, loss of cell proteostasis, telomeric shortening, cell senescence, mitochondrial dysfunction, epigenetic alterations, genomic instability, stem cell exhaustion, among others [14]. The multi-level complexity underlying the aging process involves interactions between nucleic acids and proteins and is also influenced by environmental factors, constituting the aging phenotype [15]. Metabolomics is an emerging field among the so-called “-omics sciences” and correspond to a promising tool to understand the phenotype of cells, tissues, organs, and organisms in different conditions [16–18]. Thus, by exploring the metabolome, set of all metabolites in complex organisms, phenotypic changes related to biological functions can be revealed, especially when subtle changes in the concentration of metabolites can be targeted [19]. Some effective studies have been conducted in this context, unveiling the impact of aging on different tissues [15]. By exploring the aging physiology, the course of diseases in which the incidence increases with age, such as Alzheimer's disease, rheumatic disorders, cardiovascular, metabolic diseases, and, a prominent example, cancer, can be understood [20].

In the present study, we investigated the altered metabolic pathways in dental follicles of third-molar teeth from young and adult individuals by an untargeted metabolomics approach. We aimed to provide a list of predicted altered pathways, which can pave the way for further research that may use dental follicles/dental sacs as a model to understand aging, tooth eruption, bone physiology, and epithelium-mesenchymal interactions, as well as other phenomena that can be assessed in this peculiar tissue.

## Materials and Methods

### Patients and Tissue Specimens

A convenience sample (*n* = 20) of formalin-fixed paraffin-embedded (FFPE) tissue samples was retrieved from the archives of the Oral Pathology Service at *Universidade Federal de Minas Gerais* (UFMG, Brazil). Dental sacs associated with impacted/completely unerupted mandibular third molars with diagnosis confirmed by the histopathological analysis were used. The histopathological diagnosis of all samples was confirmed by three investigators (RSG, CCG, and VCB). After searching the files and retrieving the samples from the archives, we sought to select the best cases from the youngest and the oldest patients possible, based on tissue availability as well as histological features. Thus, samples showing intense inflammatory infiltrate or bony trabeculae were excluded since both could potentially affect the findings and/or metabolite extraction protocol. Samples with the clinical diagnosis of odontogenic cysts were also excluded.

### Sample Preparation

Briefly, ~10 mg of tissue was obtained from FFPE specimens. Thick tissue sections of 20 μm were obtained using a manual microtome and collected into previously weighed microfuge tubes. The first two sections were discharged to remove potential surface contaminants. Tissues were then deparaffinized by four sequential incubations with cold xylene (Merck, Darmstadt, HE, Germany), each followed by centrifugations (15,000 × *g*, 15 min, 4°C). Residual solvent was dried using a dry bath incubator at 37°C and then weighed. The metabolite extraction solution was composed of a mixture of HPLC grade methanol:water:chloroform (3:1:1, v/v/v) (Sigma-Aldrich, San Luis, MO, USA). The volume of extraction solution was normalized *per* biomass of each tissue pellet (1 mg:20 μL of solution). Samples were homogenized using an ultrasonic bath for 10 min and centrifuged. The supernatant was filtered in nylon syringe-filters (13 mm diameter, 0.22 μm pore size), collected into inserts in glass vials, and sealed after sample preparation. A quality control (QC) sample was prepared by pooling 10 μL of each sample. Samples were stored at −80°C until analysis.

### High-Performance Liquid Chromatography-Mass Spectrometry

Extracts were examined by HPLC-MS (Shimadzu HPLC System, LC 20A, Kyoto, Japan) coupled with a Bruker Quadrupole Time-of-Flight (QToF) mass spectrometer (Bruker micrOTOF QII, Billerica, MA, USA). A reverse phase, C_18_, 5 cm × 2.1 mm i.d., 1.9 μm chromatography column (Supelco Discovery HS, Bellefonte, PA, USA) was used for metabolite separation at 40°C. The HPLC was operated according to the following parameters: injection volume = 10 μL; flow rate = 0.3 mL/min. HPLC solvents were taken as follows: A, MilliQ Water with 0.1% (v/v) formic acid; and B, acetonitrile (ACN, Sigma-Aldrich, San Luis, MO, USA) with 0.1% (v/v) formic acid (Sigma-Aldrich, San Luis, MO, USA) for positive ionization mode; and A, MilliQ water; and B, ACN for negative ionization mode. The separation was conducted with gradient mode, in which the elution range was given as follows: 25–95% B (20 min); 95% B (3 min); 95–20%B (2 min); and 20% B for 5 min. Mass spectra were acquired using positive and negative-mode electrospray ionization (ESI+ and ESI-, respectively). The capillary voltage was 4,500 V for ESI+ and 3,500 V for ESI-. Nitrogen was used as cone and desolvation gas with a pressure of 2 bar and a flow rate of 7.0 L/h. The source temperature was 100°C, and the desolvation temperature was 180°C. Nitrogen was also used as collision gas and it was generated by a nitrogen generator (NM32LA, Peak Scientific, SP, Brazil). A full scan from 90–1200 *m/z* was obtained using sodium formate as calibrator. The QC sample was reinjected multiple times after each five sequential sample injections.

### Data Preprocessing

Raw data were converted into .mzXML open format with MSConvert software (ProteoWizard, v.3.0). After conversion, optimization of parameters for data treatment was performed using QC data files. Data were optimized separately for ESI+ and ESI- in IPO package version 1.16.0 (Libiseller et al., 2015) in R Statistic workplace (R version 3.6.2). A two-step workflow was employed: the first consisted of optimization of initial default XCMS parameters followed by optimization of the whole set of parameters for *peak picking* and *retgroup* functions. We edited the *minfrac* and *minsample* parameters (set to 0.7 and 2, respectively), since they contribute to the concordance of *m/z* features within each group, increasing analytical robustness [21]. Optimized values of each parameter are shown in Supplementary File 1. Files were then processed on the XCMS package [22] in R Statistical workplace. Features extracted at <2.5 min and >20 min were removed. Duplicity was also checked by Pearson's correlation of features coeluted within a 2.5 min interval, considering a 5 ppm mass error (for this purpose, *r* > 0.7 correlation of peak intensities were considered as features duplicities and were removed from the data matrix). After this preprocessing step, the data matrix consisted of 3,530 and 475 *m/z* features, in ESI+ and ESI- modes, respectively.

### Data Processing and Statistical Analysis

Multivariate analysis was conducted on MetaboAnalyst 4.0 online platform [23, 24]. Principal Component Analysis (PCA) and Partial Least Square Discriminant Analysis (PLS-DA) models were constructed based on data input after data filtering and missing value imputation. Features presenting more than 20% of missing values were excluded, and the residual missing values were replaced by the half of minimum value of intensity for each feature; data filtering based on relative SD (features presenting RSD > 25% for the peak intensities in the QCs were excluded) and interquartile range (IQR), eliminating features with poor analytical consistency and non-informative variables. Data were then median normalized, log-transformed, and Pareto-scaled for multivariate analysis. For univariate statistical analysis, the same filters and normalizations were applied except for the Pareto scaling. Shapiro-Wilk normality test, followed by Independent Samples *t*-Test (Welch's *t*-Test) (accounting for Levene's test for equality of variances) or Mann-Whitney U Tests were conducted on IBM SPSS Statistics 26 (IBM Corporation, v.26.0.0.0) when applicable. False Discovery Rate (FDR) was applied to employ the Benjamini-Hochberg method and a cutoff of 5% was considered.

### Pathway Enrichment

As a functional interpretation of global metabolomics data can be challenging [25], we used the MetaboAnalyst 4.0 module called “MS Peaks to Pathways,” which integrates *mummichog* and Gene Set Enrichment Analysis (GSEA) algorithms to predict changes in metabolic pathways in given conditions [26]. *Mummichog* [27] is an algorithm based on Over Representation Analysis (ORA), which assumes that a certain degree of random errors during individual peak assignment will not change the collective behavior jointly determined by all metabolites involved in the pathways [28]. This algorithm tests if statistically significant peaks of a given list are enriched compared with null models drawn from the user input list. GSEA [29] is a cutoff-free method that evaluates the overall differences between two distributions based on Kolmogorov–Smirnov tests and favors the detection of subtle and consistent alterations that can be lost by ORA approaches.

For this purpose, a five-column table was built consisting data of 2,068 remaining features retained after data filtering of MetaboAnalyst for both ionization modes. The input table presented the headers “*m.z*” for mass-to-charge ratios; “*r.t*” for retention time; “*p-values*” from both Independent Samples *t*-Test or Mann-Whitney *U* Test (raw *p*-values, non-FDR corrected); “*mode*” indicating the ionization mode (*positive* or *negative*); and “*t.score*” calculated for each feature. A mass error of 10 ppm was used since it seemed more reasonable with QToF mass resolution, as *Ms peaks to Pathways* developers encourage inputs derived from high-resolution analytical platforms, such as Orbitrap or other Fourier transform mass spectrometry family. For *mummichog* analysis, a *p*-value cutoff of 0.05 was used. The library selected was the Human MFN Model, which is manually curated and originated from different sources (KEGG, BiGG, and Edinburgh Model) [30].

## Results

### Sample Characterization

**Figure 1:**
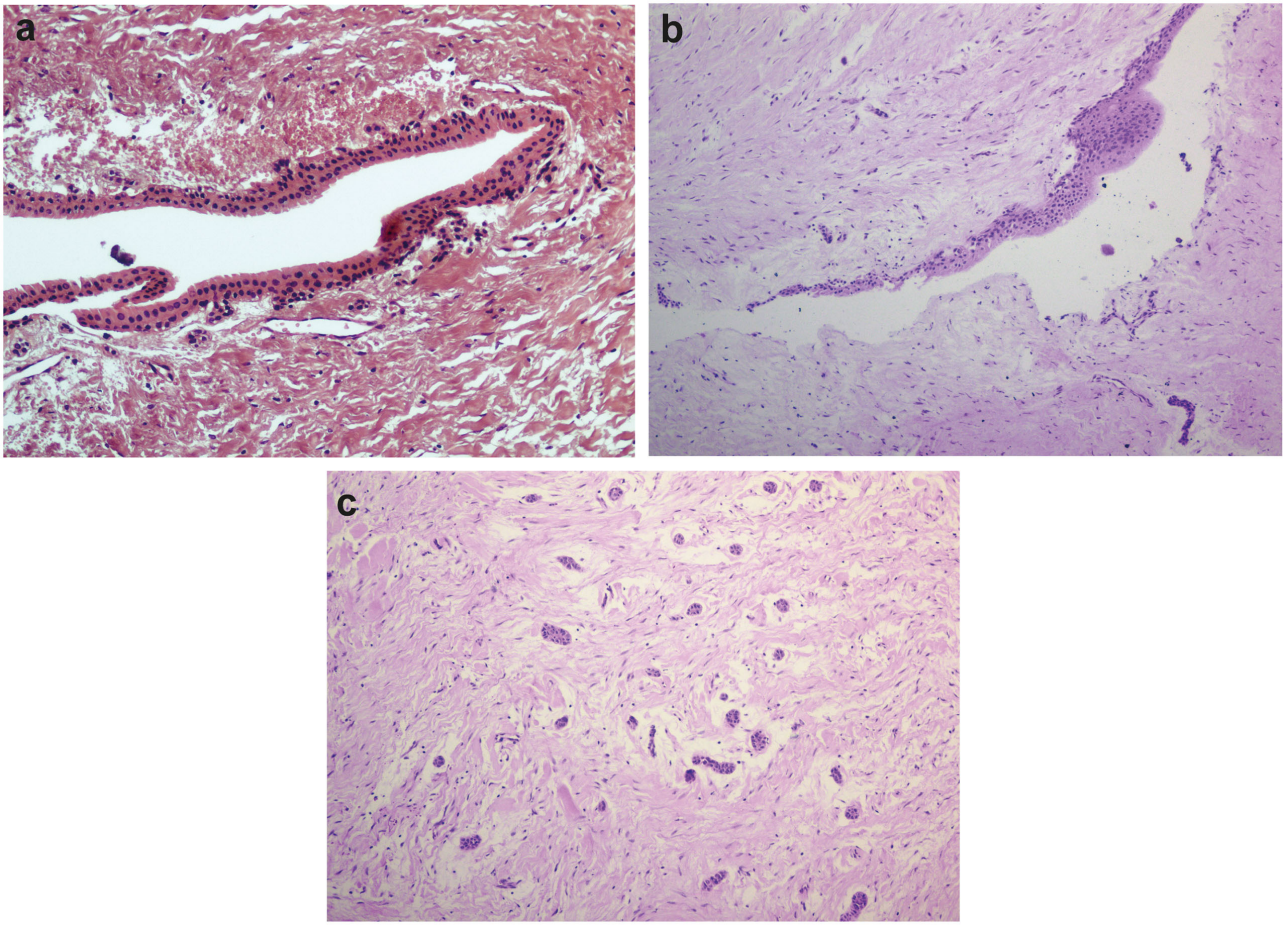
Histological features of dental follicles associated with unerupted mandibular third molars. **(a)** Reduced enamel epithelium lining connective tissue of dental follicle (x20 magnification). **(b)** Stratified squamous epithelial lining with focal thickening (x10 magnification). **(c)** Typical islets of the odontogenic epithelium were commonly observed in dental follicles from young individuals (x10 magnification). Slides were hematoxylin-eosin stained.

Thirteen samples were included in the young group of patients (13–16 years old, median = 15 years old), and seven in the adult group (26–38 years old, median = 28 years old). Although we originally intended to have equal numbers of samples in both groups, samples from dental follicles of older patients were difficult to obtain, as most of the surgical procedures to remove unerupted thirds molars occur in adolescents. The young group was comprised of six boys and seven girls. Four females and three males formed the adult group. The main histological findings are shown in Figure 1. Not all samples exhibited evident epithelial lining, but the presence of islets of the odontogenic epithelium was frequently observed, especially among younger individuals (Figure 1c). We observed the presence of reduced enamel epithelium in 6/13 samples in the young group of patients. Squamous epithelium was present in 3/7 samples among adult individuals and was also present in focal areas of 2/13 samples from young patients.

### Quality Assessment and Statistical Evaluation

Principal Component Analysis indicated good analytical stability during the HPLC-MS run, for both positive and negative ionization modes (Supplementary File 2). PLS-DA analysis showed some degree of group separation, but the generated models could not be interpreted as predictive and were considered overfitted (Supplementary File 2). The univariate analysis resulted in a total of 287 molecular features statistically significant (*p* < 0.05, non-significant after FDR correction) from a total of 2,068 features in both positive and negative ionization modes. Only two *m/z* features were retained after 5% correction of FDR (*m/z* 647.2999, adjusted *p*-value < 0.0001; *m/z* 379.1334, adjusted *p*-value < 0.0001, both detected on ESI+ mode). Since a single *m/z* feature can match multiple compounds [27], they were not individually examined.

### Pathway Enrichment

**Figure 2:**
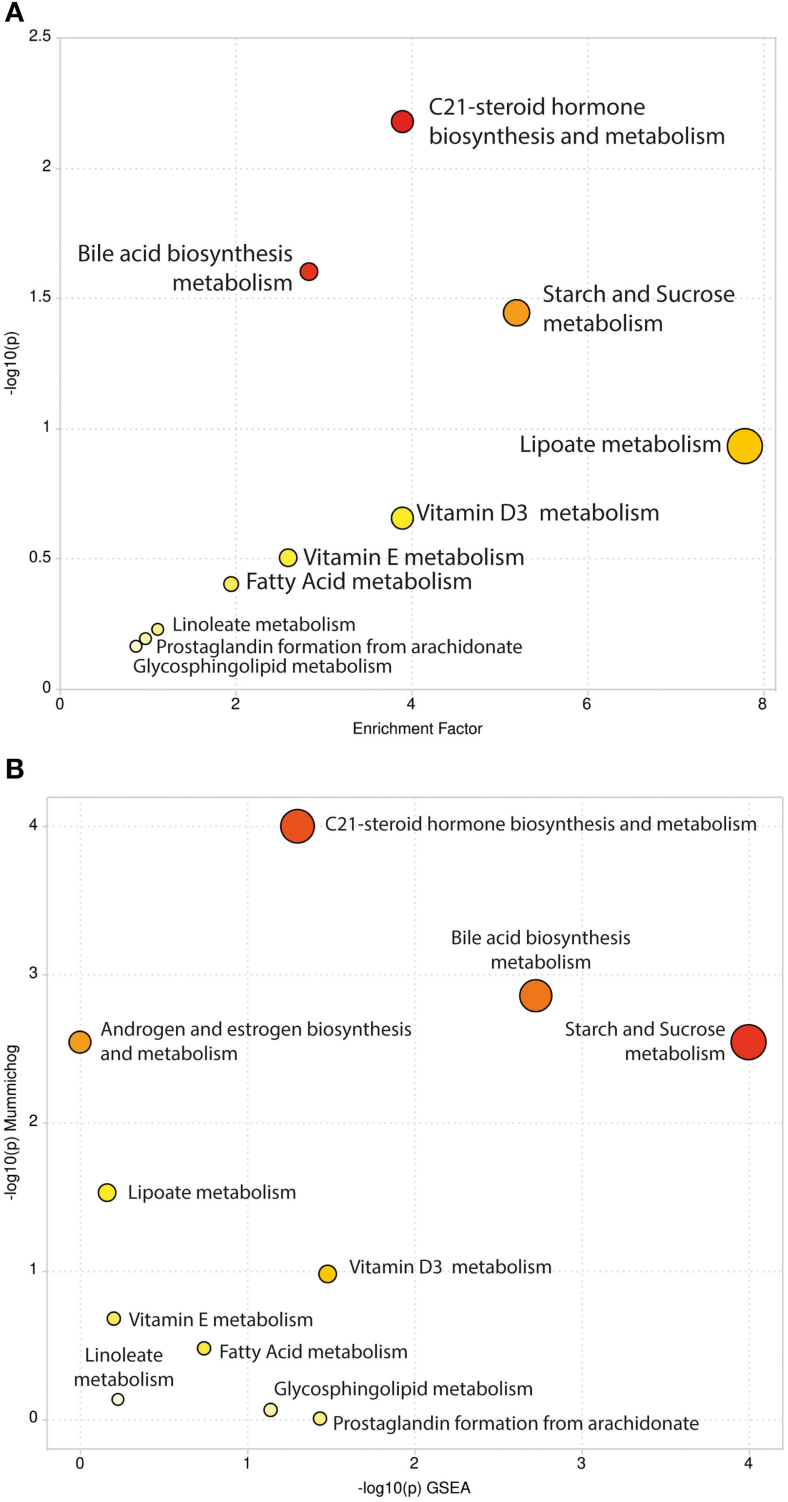
Scatter Plots of Pathway Enrichment Analysis provided by *mummichog* **(A)** and by its integration with GSEA **(B)**. The color and size of each circle correspond to its *p*-value and enrichment factor, respectively. Darker tones indicate more statistically relevant predicted pathways. The size of each dot represents the ratio between significant pathway hits and the expected number of compound hits within the pathways.

We carried out pathway enrichment based on *mummichog* and its integration with the GSEA approach, currently available within the “MS peaks to pathways” module of MetaboAnalyst 4.0. It is important to emphasize that the prediction algorithms were solely used as tools to identify metabolic pathways, with no purpose of annotating metabolites. *Mummichog* v.2 was used, and it includes retention time information to increase the confidence and robustness of potential compound matches [30], thus resulting in 248 matched compounds (Supplementary File 3). By executing the same analysis without retention time information (*mummichog* v.1), 1,355 compounds were matched and resulted pathways were reported (Supplementary File 4). For the sake of clarity, the discussion will be based on the results of the analysis that accounted for retention time analysis, although we cannot entirely discharge any other results. This analysis identified several pathways, including the C-21 steroid hormone biosynthesis, the bile acid biosynthesis, the galactose metabolism, the androgen and estrogen metabolism, the starch and sucrose and lipoate metabolic pathways (Gamma-*p*-values < 0.05) in *mummichog* approach. Tables 1, 2 provide the results from the *mummichog* approach alone and integrated with GSEA, respectively. Figure 2 shows scatter plots derived from each analysis.

**Table 1:** Results of the *mummichog* pathway analysis.

**Enriched pathways**	**Pathway total**	**Hits.total**	**Hits.sig**.	**Expected**	**FET**	**EASE**	**Gamma-*p* value**
C21-steroid hormone biosynthesis and metabolism	8	8	4	1.0256	0.0066	0.0423	0.0436
Bile acid biosynthesis	11	11	4	1.4103	0.0250	0.1016	0.0443
Galactose metabolism	3	3	2	0.3846	0.0360	0.2956	0.0447
Androgen and estrogen biosynthesis and metabolism	3	3	2	0.3846	0.0360	0.2956	0.0447
Starch and Sucrose Metabolism	3	3	2	0.3846	0.0360	0.2956	0.0447
Lipoate metabolism	1	1	1	0.1282	0.1171	1	0.0477
Vitamin D3 (cholecalciferol) metabolism	2	2	1	0.2564	0.2214	1	0.0522
Vitamin E metabolism	3	3	1	0.3846	0.3141	1	0.0569
Fatty acid metabolism	4	4	1	0.5128	0.3964	1	0.0616
Linoleate metabolism	7	7	1	0.8974	0.5914	1	0.0766
Prostaglandin formation from arachidonate	8	8	1	1.0256	0.6420	1	0.0818
Glycosphingolipid metabolism	9	9	1	1.1538	0.6868	1	0.0871

*The results of this module consist of the total number of hits, the raw p-value (Fisher's Exact Test, FET and EASE, its more conservative version), and Gamma-adjusted p-value, which means Fisher's p-values calculated using the list of significant features, adjusted for the null distribution of permutation p-values. “Pathway total” represents the total number of empirical compounds in the pathways, and “hits total” the number of empirical compounds hits from input data of users. “Expected” means the expected number of empirical compound hits in the pathway*.

**Table 2:** Integrated results of the *mummichog* and Gene Set Enrichment Analysis (GSEA) Pathway.

**Enriched Pathways**	**Total Size**	**Hits**	**Hits Sig**.	**Mummichog *p* values**	**GSEA *p* values**	**Combined *p* values**
Galactose metabolism	3	3	2	0.0360	0.0714	0.0179
Starch and Sucrose Metabolism	3	3	2	0.0360	0.0714	0.0179
C21-steroid hormone biosynthesis and metabolism	8	8	4	0.0066	0.4362	0.0198
Bile acid biosynthesis	11	11	4	0.0250	0.1979	0.0313
Androgen and estrogen biosynthesis and metabolism	3	3	2	0.0360	0.7143	0.1201
Vitamin D3 (cholecalciferol) metabolism	2	2	1	0.2215	0.2698	0.2281
Lipoate metabolism	1	1	1	0.1172	0.5192	0.2312
Prostaglandin formation from arachidonate	8	8	1	0.6421	0.125	0.2827
Fatty Acid Metabolism	4	4	1	0.3964	0.4286	0.4711
Vitamin E metabolism	3	3	1	0.3141	0.6857	0.5461
Glycosphingolipid metabolism	9	9	1	0.6868	0.3656	0.5981
Linoleate metabolism	7	7	1	0.5914	0.5568	0.6951

*GSEA considers the overall ranks of features without using a significant cutoff. The module uses Fisher's method to combine raw p-values of mummichog and GSEA approaches*.

## Discussion

In the present study, we aimed to assess the metabolic alterations related to aging in dental sacs of impacted or unerupted mandibular third molars. These are the teeth that are the most frequently associated with odontogenic lesions development [13, 31] and are the last tooth to achieve occlusal plane, generally erupting between 17 and 26 years [13]. Considering that most of the third molar extraction surgeries are performed at a younger age [32, 33], it was difficult to retrieve dental follicle samples from patients above 30 years old. We observed the presence of reduced enamel epithelium in 6/13 samples in the young group of patients. Typical small islets and strands of odontogenic epithelium were most commonly observed in the young group, agreeing with the literature [7]. Squamous epithelium was present in 3/7 samples among adult individuals. If squamous metaplasia of the reduced enamel epithelium can represent an early sign of pathological change in dental follicles is debatable [8–12, 34], as tissues can naturally undergo morphological changes to adapt to new circumstances [7, 35–38]. The relevance of this debate relies on the absence of a consensus in the clinical practice guidelines justifying prophylactic surgical removal of asymptomatic disease-free impacted teeth [13].

Differences between the young and adult dental follicle groups were observed in many metabolic pathways, such as the C21-steroid hormone biosynthesis and metabolism, the bile acid biosynthesis, the galactose metabolism, the androgen and estrogen biosynthesis and metabolism, the starch and sucrose metabolism, and the lipoate metabolism. Although we were not able to observe which metabolic pathways had decreased or increased activities between experimental groups, these metabolic pathways were already described in the aging context and will be briefly discussed. It is important to point out that puberty is, likewise, a complex process coupled to several hormonal and physiological changes [39], which must be kept in mind when dealing with metabolomic studies assessing young patients and may explain, for example, the fact that we found differences in androgen and estrogen biosynthesis and metabolism between our experimental groups. We encourage further validation studies on larger sample size and based on targeted approaches.

C21-steroid hormone biosynthesis and metabolism comprises progesterone-aldosterone and progesterone-cortisol/cortisone axes that mediate a wide variety of biological processes such as reproductive function, sexual development, electrolyte balance, blood pressure, and stress responses [40]. Steroidal metabolome was previously demonstrated to be influenced by sex, age, and circadian cycle [40–42].

Bile acids (BAs) comprise a group of important physiological agents for cholesterol metabolism, intestinal nutrient absorption, biliary secretion of lipids, toxic metabolites, and xenobiotics [43]. Through activation of signaling pathways triggered by the activation of G-protein-coupled receptors (GPCRs) or nuclear bile acid receptors (including farnesoid-X receptor, FXR, also known as NR1H4), bile acids have been shown to regulate not only their own synthesis and enterohepatic recirculation, but also regulate triglyceride, cholesterol, energy, and glucose homeostasis [44]. Changes in serum and blood plasma levels were already found to be age-related and sex-specific [43, 45]. Interestingly, genes related to the bile secretion pathway were reported to act in tooth germs development of rats at RNA and protein levels [46].

The main pathway of galactose metabolism in humans involves the conversion of galactose into glucose by galactokinase and galactose-1-phosphate uridyl-transferase for glycolysis [47, 48]. In animal models, long-term D-galactose exposure induces the acquisition of an aging phenotype, which has been recognized as being similar to those in naturally aged mice and rats [49]. Age-induced animals exhibit increased reactive oxygen species (ROS) formation and decreased antioxidant enzyme activity in the brain, poor immune responses, cognitive dysfunctions, weakened motor function, and shortened lifespan [48, 50], mainly the effects of the accumulation of senescent cells in naturally aged organisms [20, 51–53]. In these models, the impacts of impaired galactose metabolism have already been studied on the brain, liver, lungs, heart, kidney, skin, reproductive systems, and others [54]. Other carbohydrates cause oxidative stress by activation of mitochondrial metabolism of glucose, leading to ROS generation [55]. In this context, starch and sucrose metabolism may also be related. In this case, ROS is generated through mitochondrial respiratory chain enzymes, xanthine oxidases, lipoxygenases, cyclooxygenases, nitric oxide synthases, and peroxidases [55].

Lipoate metabolism plays a key role in mitochondrial functions [56]. Lipoate is a covalently bound cofactor essential for five redox reactions in humans: four 2-oxoacid dehydrogenases and the glycine cleavage system (GCS). Two enzymes are derived from the energy metabolism, α-ketoglutarate dehydrogenase and pyruvate dehydrogenase; and three are derived from the amino acid metabolism, branched-chain ketoacid dehydrogenase, 2-oxoadipate dehydrogenase, and the GCS [57]. Lipoate is the conjugate base of lipoic acid (LA), and the most prevalent form of LA under physiological conditions. It presents a highly negative reduction potential, increases the expression of antioxidant enzymes, and participates in the recycling of vitamins C and E. Due to these properties, LA is called the “universal antioxidant” [58]. LA displays anti-apoptotic and anti-inflammatory properties in *in vivo* and *in vitro* studies. Importantly, it has been shown that LA reverses the age-associated loss of neurotransmitters and their receptors, which can underlie its effects on cognitive functions [59].

Cellular, genetic, endocrine, molecular, and environmental factors were also involved in tooth eruption (as we are dealing with unerupted and impacted teeth) and were not entirely covered in our study design. Nutritional status, body mass index (BMI), socioeconomic status, and others should be stratified in further studies with larger sample size, since these are important factors affecting the metabolome and, accordingly, tooth eruption [60–63]. Moreover, causes of primary failure of eruption or delayed tooth eruption are not fully understood, but disruptions and/or total failure in the ability of dental follicles leading to bone resorption throughout the gubernacular canal are discussed [60, 61, 64]. From this perspective, metabolic alterations in dental follicles may lead tooth eruption to fail and should be explored.

The more deeply we understand tissue physiology, the more we become capable of reframing several biological questions, changing the *status quo*, and casting light into new perspectives. Recently, from genetic approaches, it has been observed that normal, pathology-free tissues also can harbor pathological mutations, some of which are known oncogenes, implicating a new way to look at genetic alterations [65–67]. Benign tumors often can exhibit hotspot mutations and still present indolent clinical behavior [67–69]. Regarding dental follicles, no hotspot mutations were found until now [70]. Global profiling of DNA methylation and hydroxymethylation was also recently explored, and, despite age-related decrease of global DNA hydroxymethylation found, the biological meaning of this epigenetic profile change in dental follicles remains to be elucidated [71].

Genotype-phenotype interactions are complex, and many variables can elicit different responses at cells, tissues, and organisms. Metabolomics can bridge the gap, making paradoxes to be reconciled. Recently, metabolism from a wide variety of sources (e.g., alcohol and microbial metabolism) was found to modify DNA and histones and exerts specific effects on cell biology, systemic physiology, and pathology [72]. Polyphenism, a peculiar sub-type of phenotypic plasticity present in several animal species were ultimately proposed to happen in humans at a metabolic level [73]. In our study, the C21-steroid hormone biosynthesis, the bile acid biosynthesis, the galactose metabolism, the androgen and estrogen biosynthesis, the starch and sucrose and lipoate metabolism have been found to correlate to aging in dental sacs.

It is important to note that our predictions of altered metabolic pathways were performed based on untargeted HPLC-MS-based metabolomics of FFPE tissue samples, which has just recently become appreciated in the metabolomics field, inspiring protocols to be developed and optimized [74–76]. In this provocative and hypothesis-generator study, we shed light on few aspects of dental follicles physiology and so many others must be discovered. Dental follicles of unerupted/impacted teeth (i.e., dental sacs) are a unique tissue in the human body, hermetically encased within the alveolar bone. They are less exposed to exogenous environmental agents and are a reminiscence of tissue interactions dating back to early odontogenesis. We encourage scientists to consider this valuable tissue as a unique model and to explore its potential to answer a wide range of questions in the fields of physiology, developmental biology, and pathology.

## Data Availability Statement

The original contributions generated for this study are included in the article/Supplementary Material, further inquiries can be directed to the corresponding author/s.

## Ethics Statement

The studies involving human participants were reviewed and approved by Ethics Committee of Universidade Federal de Minas Gerais (UFMG, Brazil) (protocol number/approval: 30697120.9.0000.5149/4.082.478). Written informed consent to participate in this study was provided by the participants' legal guardian/next of kin.

## Author Contributions

VB, JV, TP, FD-A, YL, and VM performed the experiments. VB, JV, RM-C, and FL-L performed the bioinformatic analyses. VB, JV, and RG performed data analyses. VB drafted the manuscript and generated the text and figures. LS and LL provided resources for HPLC-MS instrumentation. AM, GC, CG, and RG critically revised the manuscript. RG and CG provided leadership for the project. All authors contributed to the final manuscript.

## Conflict of Interest

The authors declare that the research was conducted in the absence of any commercial or financial relationships that could be construed as a potential conflict of interest.
