# Supplementary material for: Age-Related Metabolic Pathways Changes in Dental Follicles: A Pilot Study
Source: Front Oral Health. 2021 Jun 18;2:677731. doi: 10.3389/froh.2021.677731 (PMC8757705; doi:10.3389/froh.2021.677731)
Supplement: Supplementary file 1 [file Data_Sheet_1.zip › Supplementary Materials - 677731/Supplementary file 2.PDF]

Supplementary File 2

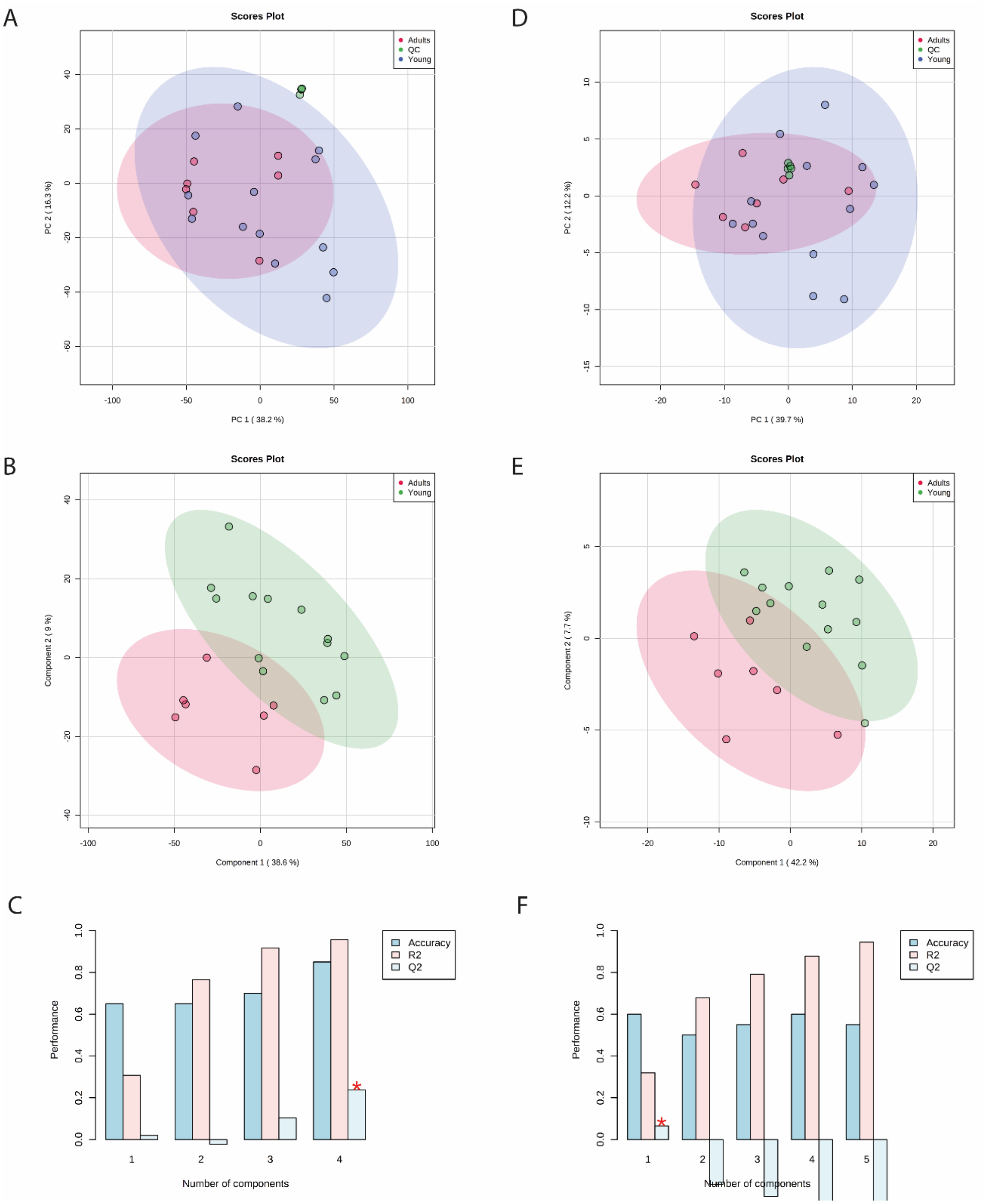

Principal Component Analysis (PCA) and Partial Least Square Discriminant analysis (PLS-DA). The explained variances are shown in brackets (A, B, D, E), for positive (A-C) and negative ionization modes (D-F). Grouping of QC samples (green dots in PCA plots) indicates a good analytical stability (A and D). Even though both PLS-DA (B for ESI+ and E for ESI-) suggests differences between the groups, R2 and Q2 were poor (C and F, for ESI+ and ESI-, respectively). Plots were generated in MetaboAnalyst 4.0.
